# Peer review: Risk and risk tolerance

**DOI:** 10.1371/journal.pone.0273813

**Published:** 2022-08-26

**Authors:** Stephen A. Gallo, Karen B. Schmaling

**Affiliations:** 1 Scientific Peer Advisory and Review Services Division, American Institute of Biological Sciences, Herndon, Virginia, United States of America; 2 Department of Psychology, Washington State University, Vancouver, Washington, United States of America; Chinese University of Hong Kong, HONG KONG

## Abstract

Peer review, commonly used in grant funding decisions, relies on scientists’ ability to evaluate research proposals’ quality. Such judgments are sometimes beyond reviewers’ discriminatory power and could lead to a reliance on subjective biases, including preferences for lower risk, incremental projects. However, peer reviewers’ risk tolerance has not been well studied. We conducted a cross-sectional experiment of peer reviewers’ evaluations of mock primary reviewers’ comments in which the level and sources of risks and weaknesses were manipulated. Here we show that proposal risks more strongly predicted reviewers’ scores than proposal strengths based on mock proposal evaluations. Risk tolerance was not predictive of scores but reviewer scoring leniency was predictive of overall and criteria scores. The evaluation of risks dominates reviewers’ evaluation of research proposals and is a source of inter-reviewer variability. These results suggest that reviewer scoring variability may be attributed to the interpretation of proposal risks, and could benefit from intervention to improve the reliability of reviews. Additionally, the valuation of risk drives proposal evaluations and may reduce the chances that risky, but highly impactful science, is supported.

## Introduction

Research funding agencies, including the US National Institutes for Health (NIH), rely on peer review to help identify the most scientifically meritorious and impactful research proposals [1]. Of concern is evidence that reviewers can discriminate good from bad proposals more reliably then good from great proposals [2–4]. Reviewers are often expected to evaluate beyond their discriminatory power, resulting in relying on biases and preferences [5,6]. Previous studies document cases of low inter-reviewer reliability and more variability between reviewers than between proposals [7–10].

However, little research has examined associations between reviewer characteristics and score variability [9–12]. A potentially important characteristic is risk tolerance, which may reflect a general personality factor [13–16]. There is evidence that reviewers evaluate risky proposals negatively, where the perceived weaknesses are not fully offset by proposal strengths [11,17,18]. Examples of such potential risks in research proposals include new or poorly constructed design or methodology, or principal investigators (PIs) transitioning to new areas. It remains unexplored whether reviewers favor differing levels of risk or sources of weakness, although it is clear that the NIH proposal evaluation criteria–approach, significance, innovation, PI, and environment–are not equally predictive of overall scores [19,20].

In this study, we conduct a cross-sectional experiment of 605 NIH peer reviewers’ evaluations of mock primary reviewers’ comments (overall impact statements (OISs)) in which the level and sources of risks were manipulated. Manipulated sources of risk were the PI or the approach and manipulated levels of risk were low or moderate, resulting in four OISs: low risk PI and low risk approach (control OIS); low risk PI and moderate risk approach (approach risk); moderate risk PI and low risk approach (PI risk); and moderate risk PI and moderate risk approach (PI-approach risk). We evaluated the association of proposal risks and reviewer characteristics, including risk tolerance (see Methods), with reviewers’ criteria and overall scores.

## Methods

### Design

This study recruited reviewers experienced in NIH and NIH-style grant evaluation to participate in an on-line experiment and survey. The survey involved answering self-report measures of demographics; grant review experience, expertise, and evaluative bias; and a personality measure of risk tolerance. The experiment involved rating two basic science NIH-style overall impact statements (OISs) in random order. Participants all rated a positive OIS that had no weaknesses in any criteria category (significance, innovation, approach, investigator, environment) and a second OIS. The second OIS was randomly determined to reflect one of three combinations of weaknesses: weaknesses in the approach only; weaknesses in the PI only; weaknesses in both the approach and PI.

### Participants

**Fig 1:**
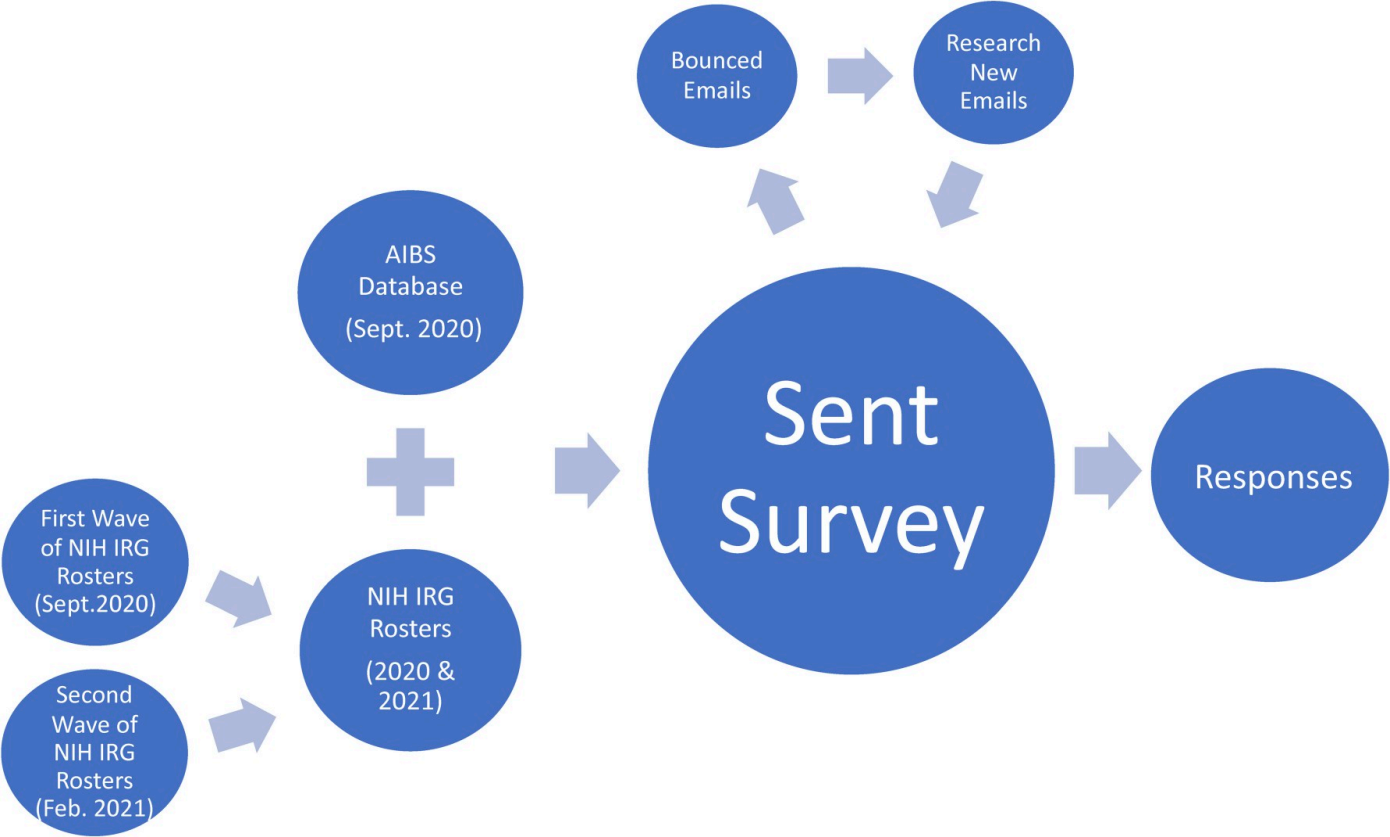
Recruitment flowchart. Recruitment flowchart detailing sample gathering from two initial sources, as well as internet searches of bounced emails. Scientists were sent the survey via email (and a reminder email) in 2 waves (September/October of 2020 and February of 2021) and responses were finalized in spring 2021.

Participants were recruited from two sources (**Fig 1**). The first source was comprised of 10,990 people in the American Institute of Biological Sciences’ (AIBS) database who had putatively reviewed for or submitted to one of the funders for whom AIBS had conducted an NIH-style peer review process. Email addresses were extracted from the database: web searches for the current email address were conducted for individuals with multiple email addresses. The second source was comprised of 1678 scientists listed on study section rosters affiliated with the following 6 (1^st^ wave) + 9 (2^nd^ wave) 2020 NIH integrated review groups (IRGs): 1^st^ wave; Cardiovascular and Respiratory Sciences; Immunology; Infectious Diseases and Microbiology; Molecular, Cellular, and Developmental Neuroscience; Oncology Basic Translational; and Vascular and Hematology; 2^nd^ wave; Biological Chemistry; Cell Biology; Digestive, Kidney, Urological Systems; Endocrinology, Metabolism, Nutrition and Reproduction Science; Genes, Genomes and Genetics; Infectious Disease; Musculoskeletal Research; Oncology Translational Clinical; and Surgical Science, Biomedical Imaging and Bioengineering. These IRGs were chosen based on size and scientific topic area; particularly, we were looking for scientists who would be reasonably familiar with the type of in vivo studies as those described in the OISs. Email addresses were identified by web searches. Only scientists affiliated with US-based institutions with US-based emails were used.

From the first source (AIBS), an initial invitation was successfully emailed to 10,594 unique individuals in three groups in September and October of 2020. There were 1430 bounced emails. The 10594 number of successful emails includes emails that were successfully emailed the first time, plus alternate email addresses found by internet research of those with bounced emails (**Fig 1**). The internet research of bounced emails also revealed people on the original list who were not pursued further if they were determined to be institutional officials, unlikely to be biomedical researchers and have conducted NIH-style peer review (e.g., emails associated with state fish and wildlife divisions), or deceased. This invitation stated how they were identified and invited them to participate if they had done one or more NIH-style reviews in the last 5 years. One reminder invitation was sent two weeks after the initial invitation to those who had not completed the survey nor opted out from further contact. Of those successfully emailed from this first source, 4.5% completed the full survey (n = 475).

From the second source (NIH), an initial invitation was emailed to the 734 unique individuals in one group in early February of 2021 with 13 bounced emails. One reminder invitation was sent two weeks after the initial invitation to those who had not opted out from further contact nor completed the survey.

Another wave was sent to 954 NIH IRG members in late February with 16 bounced emails. These numbers included those found by further email research of those with bounced addresses. From the second source, a total of 7.7% of those successfully emailed completed the full survey (n = 130).

### Measures

The measures were set up as a Qualtrics^TM^ survey in the following order, unless randomly determined as noted below. First was the consent form, which included a statement of the inclusion criteria, namely having conducted one or more NIH-style reviews in the last 5 years. Demographic information was collected next and included age, academic degrees, gender identity, English as the first language, racial/ethnic identities, and the number of study sections/reviews committees on which the participant had served in the past five years for each of several US research funding agencies (NIH, NSF, AHRQ, DoD, or other). If the participant reported serving on zero NIH, AHRQ, or DoD study sections, which all use similar review criteria and scoring procedures, they were disqualified.

OISs were rated next (see below), followed by self-reported measures of the similarity of their research relative to the OISs (on a scale of 1 to 7 where 1 is extremely similar and 7 is extremely dissimilar) and evaluative disposition (on a scale of 1 to 7 where 1 is lenient/generous and 7 is firm/rigorous). Finally, as a proxy for reviewer risk tolerance [13–15], participants completed the 13-item Openness to Experience scale from the NEO-Five Factor Inventory 3 [13–15,21], which has previously established reliability, validity, and norms. The raw scores from the Openness to Experience scale were converted to normed T scores by subject gender [21], and the T scores were retained for analysis. The T score values of our participants ranged from 28 to 60 with a median of 46 (IQR of 9). Average scores were very similar across all groups of participants. Participants were given an opportunity to add comments about the study in an open text field. Finally, they were asked to provide their email address for the honoraria ($20 Amazon gift card).

### Overall impact statements

The four OISs were designed as typical primary reviewer OISs of biomedical research proposals submitted to the NIH that address a specific disease or disability. Proposed work in all of the OISs was at the in vivo level of translatability, but was represented as addressing a critical clinical need and having high potential impact to translate to a clinical setting. The descriptions of innovation and environment did not differ between the OISs, and were written to suggest a significant degree of novelty in the hypotheses, and to have an excellent supporting environment, respectively. The level of risk in the scientific approach and/or with the PI were manipulated in three of the OISs as described below. The PI’s gender was also manipulated, but these data are not examined in this manuscript. The impact statements were constructed to address each criterion–significance, innovation, approach, investigator, environment—and to have 300–400 total words per statement.

Participants received the following instructions: “You are an unassigned reviewer who hears the following summary from an assigned reviewed of an NIH R01 application. In this scenario, you do not have the ability to reference the original proposal or discuss the application with the assigned or other reviewers. Please score the overall impact and also significance, innovation, investigator, approach and environment. You may consult the score guidance table below.” Participants rated each OIS for overall merit and five criteria on the NIH scale of 1 to 9 using whole numbers (1 = exceptional and 9 = poor).

The control OIS was created to reflect an exceptional or outstanding proposal with no discernable weaknesses, i.e., low-risk approach and a low-risk PI. All participants rated the control OIS. Participants also rated one of three manipulated OISs. The three other OISs varied in the PI and/or approach risk: low approach risk and moderate PI risk; moderate approach risk and low PI risk; or moderate approach risk and moderate PI risk. For these 3 OISs, the text was held constant for the topic being investigated, the significance and innovation of the work, and the quality of the environment. The four OISs are included in the **S1 File**.

We pilot tested these OISs prior to finalizing the text of the statements. Because past research has indicated that reviewers do not discriminate good from great proposals easily [3], the control statement was intended to reflect a great proposal (score of 1–2) and the manipulated statement was intended to reflect a good proposal (score of 3–5). We tested draft statements with 25 experienced peer reviewers. The pilot data suggested that our manipulations of risk were successful: overall scores for the control statement were significantly better than those of manipulated statements. The pilot subjects were also interviewed and based on their feedback, the OISs were modified slightly. The pilot study and the study reported here were reviewed by the Washington State University Office of Research Assurances (Assurance# FWA00002946) and granted exemptions from IRB review consistent with 45 CFR 46.104(d)(2).

### Data manipulation

Data were exported out of Qualtrics^TM^ into comma-separated format to Excel^TM^. Some variables were re-coded for the regression analyses. Most demographic data variables that were categorical in nature were coded as 0 or 1 (except for gender). These variables included race (coded as 0  =  White, 1  =  non-White), gender (1 = female, 2  =  male, 3 = non-binary), English as a first language (1 = yes, 0 = no), PhD degree (1 = yes, 0 = no), and MD degree (1 = yes, 0 = no). The measure of risk tolerance (NEO Openness to Experience) was z-score transformed, because of their large range.

### Statistical analysis

Data were imported from Excel to the statistical program R (https://www.r-project.org), and analyses were performed using the ordinal, rcompanion, and ggplot2 packages. Median values and distributions of overall and criteria scores were examined for the four OISs using violin plots. In some cases, non-parametric tests (e.g. Mann-Whitney or Kruskal–Wallis tests) were conducted to confirm differences between risk scenarios using StatPlus^TM^ software. To examine how well participants could detect risk, receiver operating characteristic (ROC) curves were calculated for the overall score using a series of thresholds over the full scoring range (1–9). We defined a true positive as a participant identifying correctly that a scenario is risky by scoring worse that the threshold. We defined a false positive as a participant identifying incorrectly that the control scenario is risky. For each risk group, the area under the curve (AUC) and the corresponding 95% confidence intervals were calculated.

Given the ordinal values and the cross-sectional, nested nature of the OIS scoring data (two sets of scores nested within each participant), multi-level ordinal regression models were used to examine the relationship of the predictors to the dependent variables of criteria and overall scores. Specifically, cumulative link mixed models were used, fitted with the Laplace approximation and variance, which looked at fixed effects from predictor variables and random effects from participants (two sets of scores per participant). An intra-class coefficient (ICC) was derived from the variance estimate of the final regression model to assess the inter-rater consistency across all participants [22]. We also estimated Cronbach’s alpha to measure the within-rater consistency between the rating of control and risk scenarios for overall and criteria scoring.

Starting with an intercept-only baseline model, predictor variables were added in blocks and models were compared via log likelihood for successive improvements in fit. Nagelkerke-R^2^ values were calculated as well, to compare models to baseline. Interaction effects were examined as variable blocks were added to the model, and if none were observed, were not included in the proceeding models. These models were applied to the overall and individual criteria scores for each OIS risk scenario. Relative likelihood profile functions were calculated using adaptive Gauss-Hermite quadrature approximation (with 10 quadrature points) for the random effects with 95% confidence intervals based on the profile function. For model comparisons, participants with missing data were removed for consistency.

Post-hoc visualizations included LOESS linear fits of the overall score for each criterion broken down by risk scenario. Jittered scatterplots using local regression (LOESS) fitting and boxplots were also used to examine relationships between variables. Also explored were the effects of the order of OIS presentation (e.g., control first) and whether the participants were recruited from the AIBS database or from NIH rosters. Some fixed effects ordinal regression models were applied to specific risk scenarios to further analyze the data.

### Response completion and demographics

In total, 592 (AIBS) + 153 (NIH IRG list) individuals completed some of the survey. Of these 745 individuals, 140 individuals did not complete the survey; 16 stopped at the consent form; 25 at the demographic section; 99 at the OISs. Of this group of participants with incomplete data, 29% (41) did not provide demographic data. Of the 99 that provided demographic information, 30% were female, 78% were White, 76% were native English speakers, 74% had PhDs, and 68% were 20–40 years since their degree; all of these proportions were similar to the demographics of the participants with complete data in the main sample (**Table 1**). However, only 40% of participants with incomplete data had been part of 10 or more review panels in the last three years (compared to 59% from the main sample; **Table 1**) and only 70% had taken part in an NIH panel (compared to 95% in the main sample; **Table 1**). Thus, many of those who did not complete the survey did so due to limited review experience. Their exclusion is consistent with review experience as the major inclusion criteria for our study.

**Table 1:** Demographic summary of sample with no missing OIS data. Total N is 605, PI risk group (199), Approach risk group (204), PI and Approach risk group (202).

Factor		Total Proportion (N)	Proportion PI Risk (N)	Proportion Approach Risk (N)	Proportion PI and Approach Risk (N)
Gender	Male	65% (361)	65% (129)	65% (132)	62% (125)
	Female	35% (197)	35% (70)	35% (72)	38% (76)
	Non-Binary	<1% (1)	0% (0)	0% (0)	<1% (1)
English as Your First Language	Yes	71% (397)	68% (136)	70% (143)	71% (144)
	No	29% (162)	32% (63)	30% (61)	29% (58)
Race/Ethnicity	Asian	18% (102)	20% (39)	14% (29)	21% (43)
	Black/African American	1% (7)	2% (3)	1% (3)	<1% (1)
	Latinx	5% (27)	4% (7)	7% (14)	4% (9)
	Native American/Indigenous	1% (4)	0% (0)	1% (2)	<1% (1)
	White	75% (421)	80% (159)	77% (157)	74% (150)
					
Degree Type	PhD	83% (464)	80% (159)	87% (178)	85% (171)
	MD	28% (158)	31% (62)	23% (47)	29% (59)
	Neither	1% (3)	<1% (1)	<1% (1)	<1% (1)
					
Years Since Degree	Up to 20 years	20% (111)	21% (41)	22% (44)	15% (31)
	20 to 40 years	66% (371)	63% (125)	64% (131)	68% (138)
	40 to 60 years	14% (77)	15% (30)	11% (23)	14% (29)
					
Total Review Panels in last 3yrs	Up to 10	41% (228)	39% (78)	37% (76)	43% (86)
	10 to 20	37% (207)	40% (80)	42% (85)	32% (64)
	20 to 30	14% (80)	14% (27)	14% (28)	15% (31)
	30 and over	8% (44)	7% (14)	8% (16)	10% (20)
					
Review Panels–Funding Agency	NIH	95% (532)	95% (190)	96% (196)	93% (188)
	NSF	19% (104)	20% (39)	18% (36)	19% (39)
	AHRQ	4% (24)	5% (10)	4% (9)	4% (8)
	DoD	38% (214)	39% (77)	41% (84)	34% (68)
	Other	68% (380)	70% (139)	62% (126)	70% (142)

Among the 605 participants (475 from the AIBS sample, 130 from the NIH sample) who had completed the survey, 7.6% (n = 46) were missing data as follows: risk tolerance, 4; racial identity, 3; degree, 1; degree year, 16; research similarity, 24; evaluative predisposition, 8.A total of 559 participants had no missing data at all (460 from the AIBS sample and 99 from the NIH sample). In the final regression models that utilized a maximum likelihood function, the full data set (N = 605) was used, but the model comparisons utilized the reduced data sets (N = 559).

Median survey duration was 13 minutes (IQR = 11 minutes). Participant demographics are listed in **Table 1**; most participants are White male PhDs with English as their first language. The majority of participants received their most recent degree 20 to 40 years ago. Just over 95% of participants had reviewed for NIH in the last 3 years, with the majority participating in up to 20 review panels over that 3-year period. Demographics were found to be relatively consistent across participants rating the three risk scenarios (**Table 1**). These demographics are very similar to those reported by NIH of their reviewers. [23] An anonymized version of these data is available on the Open Science Framework (DOI 10.17605/OSF.IO/FU83D).

## Multi-level ordinal regression modeling

We examined variance inflation factors (VIFs) for all variables used in the model and found no case where VIF>10 (**S1 Table**), which we used as a criterion for inclusion in the regression [24]. From baseline, we added risk (a contrast variable examining either PI-only, approach-only, or PI-approach scenarios compared to the control) as a predictive factor to the model for overall score, and found a large improvement in fit compared to baseline (**Table 2)**. Demographic variables were then added as a block, then the rating of their research similarity relative to the OIS and evaluative predisposition, none of which improved the model. We then added the measure for risk tolerance (NEO Openness to Experience scale), to the model, but with no improvements in fit or variance explained. We then added the five criteria scores (significance, innovation, investigator, approach and environment) as a block to the model, and found a significant improvement in fit when the model was compared to baseline. Similar steps were taken for all criteria scoring models.

**Table 2:** Overall score–multi-level ordinal regression models made with the reduced data set for direct comparison (n = 559).

Model	Variance Across Participants	Changes in 2LL (Previous Model)	Nagelkerke R^2^
Baseline Across Participants	7E-09	0	---
Risk (R)	1.283	571.6**	0.42**
R + Demographic Variable Block (DV)	1.246	8.3	0.42**
R + DV + Research Similarity (RS)	1.249	2.2	0.42**
R + DV + RS + Pre-disposition (PD)	1.249	0	0.42**
R + DV + RS + PD + Risk Tolerance (NEO)	1.247	0.3	0.42**
R + DV + RS + PD + NEO + Criteria Scores (CS)	0.4992	896.1**	0.77**
R + DV + RS + PD + NEO + CS + R:CS Interactions	0.5619 (0.1763, 1.1649)	67.9**	0.78**

* p< 0.05

** p<0.01; 95% CI in parentheses; each successive model is compared to previous via -2LL (a fixed intercept model was used as baseline); Nagelkerke R^2^ was calculated comparing to baseline model.

## Results

### Manipulation check

**Fig 2:**
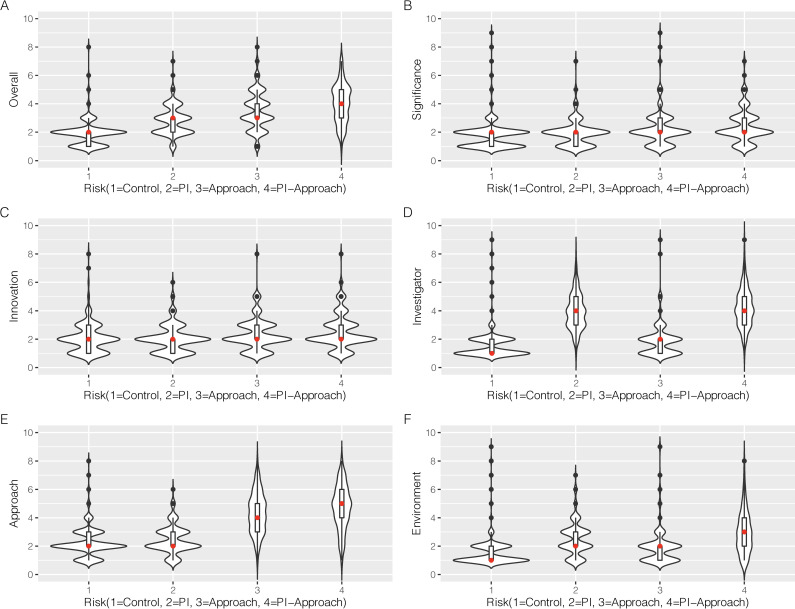
Scoring distributions. Median values (in red) and distributions of overall and criteria scores for different risk scenarios (violin plot; N = 605). A. Overall; B. Significance; C. Innovation; D. Investigator; E. Approach; F. Environment.

**Fig 3:**
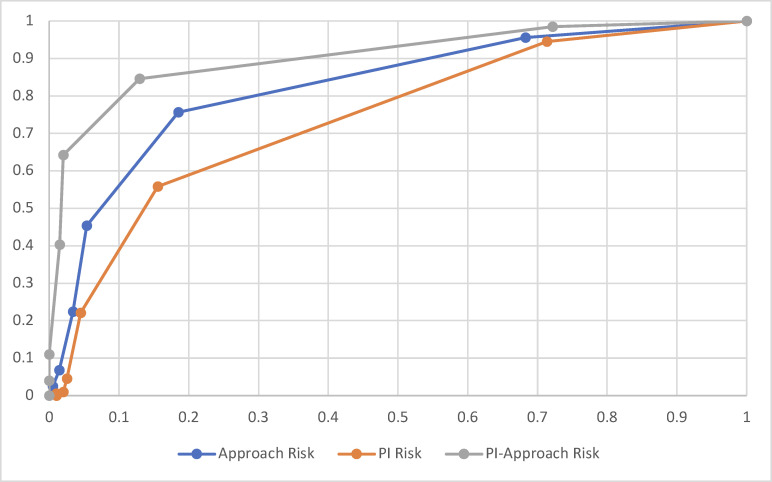
ROC plots. Receiver operating characteristic plots were calculated for the overall score using a series of thresholds over the full scoring range (1–9). Proportions of true positives versus false positives were plotted for each risk group.

The experimental manipulation was effective. Probability densities, distributions (via overlayed boxplots) and median values (in red) of the overall and criteria scores for each OIS are shown in violin plots (**Fig 2**). Lower scores are more positive, i.e., 1 = exceptional and 9 = poor. The three OISs with manipulated risk, intended to represent “good” proposals, were rated consistent with this descriptor, with less favorable scores and more dispersed scoring distributions than the control OIS, which was intended to represent a “great” proposal, and was rated in the outstanding range (**Fig 2 and S2 Table**). The median values of the overall score are similar for the PI risk and approach risk OISs, but the scores for the approach risk OIS are more dispersed and positively skewed (U = 14,113, n1 = 205, n2 = 199, p < 0.001; effect size = 0.55). The overall score of the PI-approach risk OIS was rated more mediocrely than either PI or approach risk OISs, and the scoring distribution and probability densities are dispersed. This dispersion is reflected in the ICC measurement, which across all participant groups was 0.15, reflecting the significant spread of scores within the three risk groups. While the magnitude differed across reviewers, the majority of reviewers agreed in each risk case (65%, 79% and 88% for PI, approach and PI-approach risk groups, respectively) that the manipulated OIS should receive a worse score than the control OIS. Based on the ROC plot (**Fig 3**), participants were observed to have excellent discrimination in determining the presence of risk; participants in the PI risk group had the lowest AUC (0.74 [0.70, 0.79]), with the approach risk group having an increased AUC (0.83 [0.79, 0.87]) and the PI and approach risk group having the highest AUC (0.91 [0.88, 0.94]).

Criteria scores also generally reflected the risk manipulations (**Fig 2 and S2 Table**). Significance and innovation scores had constant median values across OISs. However, the score distributions of unmanipulated criteria were affected by the manipulation of PI or approach: e.g., environment was evaluated more poorly in manipulated OISs as compared to control. Also, the PI was evaluated more poorly in the approach risk OIS than control, despite no manipulation of PI in this OIS. Moreover, approach was evaluated more poorly in the PI-approach risk OIS than in the approach risk OIS.

**Fig 4:**
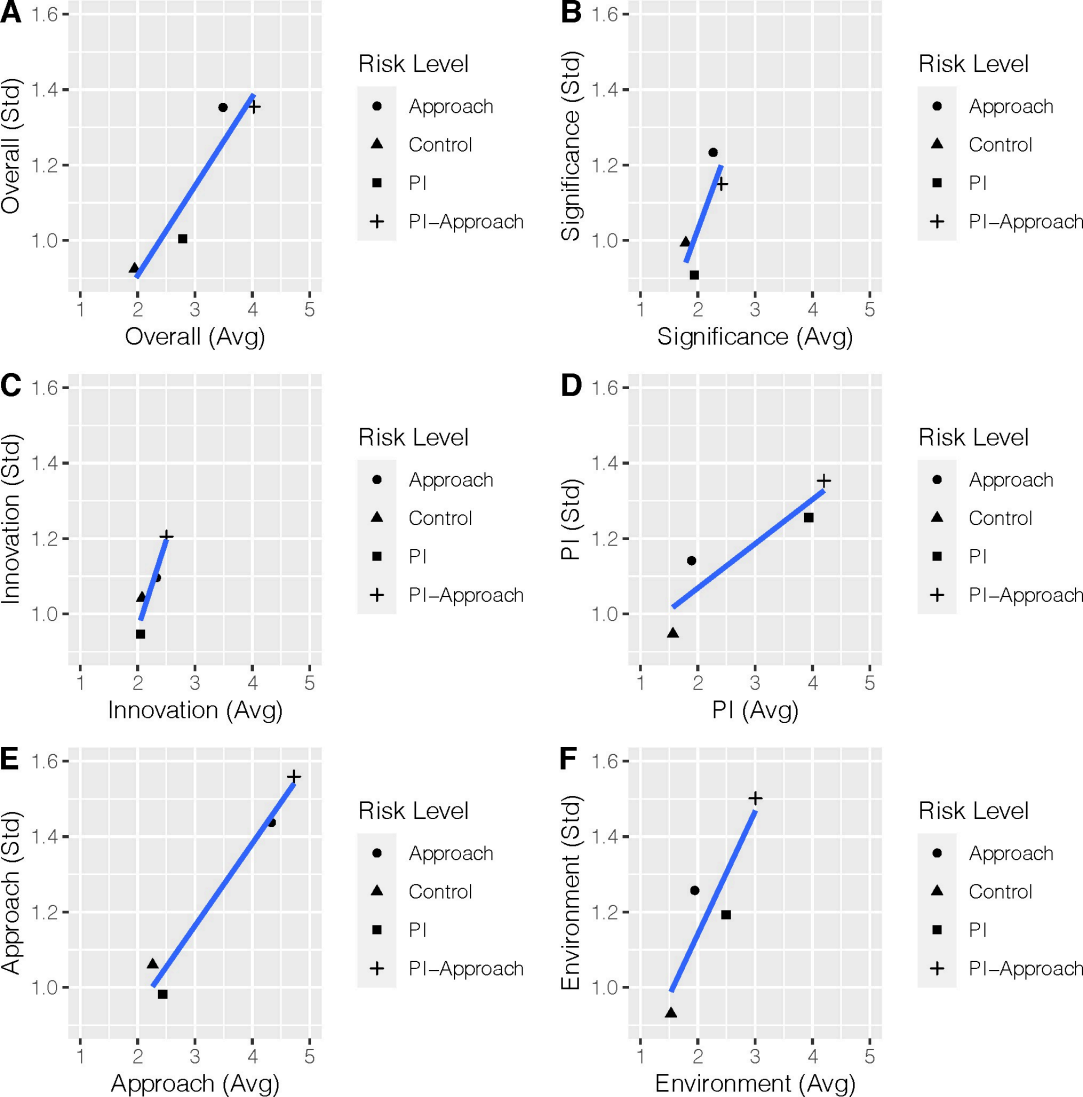
Score dispersion versus risk level. Plots of the standard deviation of scores (overall and criteria) versus mean scores for different OIS risks. A. Overall; B. Significance; C. Innovation; D. Investigator; E. Approach; F. Environment.

The risk manipulations resulted in greater dispersions in overall and criteria scores. To further examine the relationship between score dispersion and risk, mean scores were plotted against scores’ standard deviation for overall and criteria scores. There were strong associations, whereby riskier scenarios resulted in more dispersed scores (**Fig 4**).

### Multi-level ordinal regression modeling—overall score

We created a series of multi-level ordinal regression models to examine OIS risk, criteria scores and their interactions, demographic variables and risk tolerance as predictors of overall scores. The intercept of our baseline model was allowed to vary across participants; we then added blocks of variables as fixed effects and compared successive models for improvement in fit using log likelihood. Random slopes were also explored but were not found to improve model fit. To facilitate model comparisons, only participants without missing data were used (559 participants), but the final model (which utilized a maximum likelihood function and can handle missing data) used the complete data set (605 participants). The final model included interactions between the five criteria variables and risk scenario variable, with a Nagelkerke R^2^ = 0.78 (**Table 2)**. Random effects of participants accounted for a significant amount of the variance in our final model. The contributions of individual predictors are listed in **Table 3**: OIS risk, significance, innovation and approach scores, and interactions of all the criteria scores except for environment with OIS risk were significant predictors of overall scores (**Table 3**). Risk tolerance did not predict overall scores.

**Table 3:** Cumulative link mixed model of overall score fitted with the Laplace approximation from the total data set (605 participants).

Term	Odds Ratio	95% CI	p-value
Risk			
PI risk	14.98	3.40, 66.00	0.0003***
Approach risk	21.69	5.50, 85.43	<0.0001***
PI-Approach risk	36.17	8.47, 154.43	<0.0001***
Demographic Block			
Gender (Male)	1.03	0.76, 1.41	0.8411
Gender (Non-Binary)	1.05	0.05, 22.39	0.9743
Race Ethnicity (Non-White)	0.94	0.64, 1.40	0.7736
English as a First Language (Yes)	1.27	0.89, 1.81	0.1960
PhD (Yes)	0.96	0.56, 1.63	0.8711
MD (Yes)	0.91	0.59, 1.41	0.6627
Year Since Last Degree	1.00	0.98, 1.01	0.6303
Total Review Panels in the last 3 years	1.02	1.01, 1.03	0.0033**
Research Similarity	1.01	0.92, 1.09	0.9016
Evaluative Predisposition	0.92	0.82, 1.03	0.1536
NEO Openness Scale	1.02	0.87, 1.18	0.8370
Review Criteria			
Significance Score	3.53	2.60, 4.79	<0.0001***
Innovation Score	2.24	1.69, 2.97	<0.0001***
Investigator Score	0.91	0.63, 1.31	0.6213
Approach Score	3.31	2.46, 4.45	<0.0001***
Environment Score	1.15	0.80, 1.66	0.4417
Interactions			
Risk PI: Significance Score	0.84	0.49, 1.45	0.5332
Risk Approach: Significance Score	0.90	0.58, 1.39	0.6194
Risk PI-Approach: Significance Score	0.52	0.33, 0.83	0.0057**
Risk PI: Innovation Score	0.55	0.34, 0.89	0.0158*
Risk Approach: Innovation Score	0.38	0.24, 0.61	0.0001***
Risk PI-Approach: Innovation Score	0.52	0.34, 0.80	0.0026**
Risk PI: Investigator Score	2.23	1.38, 3.6	0.0011**
Risk Approach: Investigator Score	1.53	0.84, 2.79	0.1664
Risk PI-Approach: Investigator Score	1.63	1.02, 2.61	0.0409*
Risk PI: Approach Score	0.57	0.34, 0.94	0.0283*
Risk Approach: Approach Score	1.31	0.91, 1.90	0.1516
Risk PI-Approach: Approach Score	1.36	0.94, 1.98	0.1044
Risk PI: Environment Score	1.03	0.64, 1.66	0.8990
Risk Approach: Environment Score	0.75	0.44, 1.27	0.2833
Risk PI-Approach: Environment Score	0.70	0.45, 1.09	0.1113
Threshold Coefficients			
1|2	4.89	3.71, 6.07	<0.0001***
2|3	9.53	8.1, 10.95	<0.0001***
3|4	12.25	10.67, 13.83	<0.0001***
4|5	14.58	12.85, 16.31	<0.0001***
5|6	17.75	15.78, 19.72	<0.0001***
6|7	19.90	17.71, 22.09	<0.0001***
7|8	23.31	20.28, 26.34	<0.0001***

* p< 0.05

** p<0.01

*** p<0.001

### Multi-level ordinal regression modeling—criteria scores

A series of multi-level ordinal regression models was used to examine predictors of criteria scores, using a fixed-intercept baseline with random effects for participants, successively adding blocks of variables and comparing model fit. These results are presented in **S3–S12 Tables**. For all criteria score models, OIS risk was observed to significantly predict scores. Risk tolerance did not improve model fit for any criteria score other than approach for which–interacting with OIS risk—it was a statistically significant but weak predictor (see jittered scatterplot with LOESS linear fits of approach scores versus risk tolerance for different OIS risk in **S1 Fig**). Final models ranged in their fit compared to baseline models: the final innovation score model had a Nagelkerke R^2^ of 0.04 and the final PI score model had a Nagelkerke R^2^ of 0.57.

### Overall and criteria score relationships

**Fig 5:**
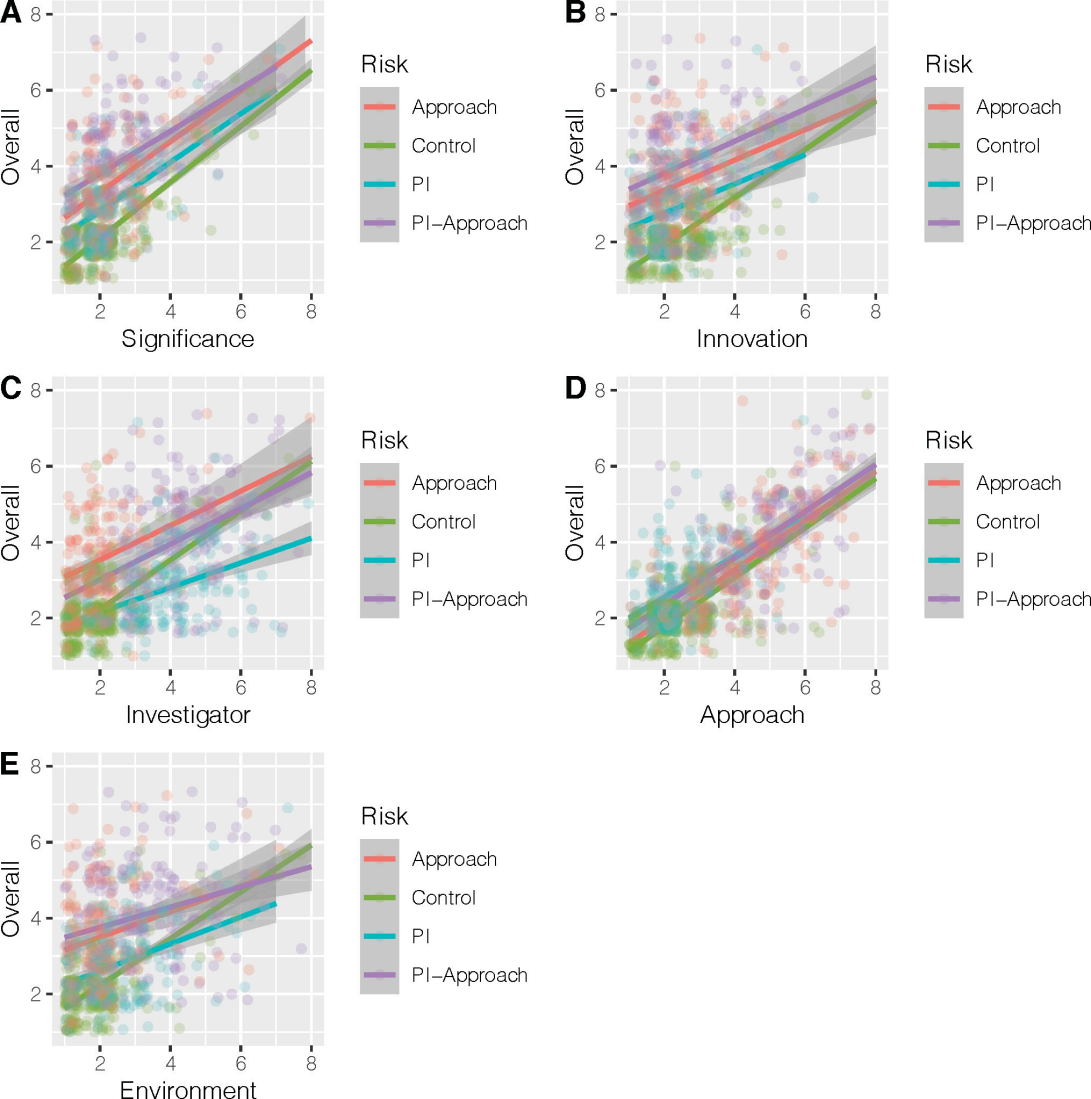
Overall versus criteria scores. Scatter plots and LOESS fits of overall score versus criteria scores for different OIS risks (Linear Fits). A. Overall vs Significance; B. Overall vs Innovation; C. Overall vs Investigator; D. Overall vs Approach; E. Overall vs Environment.

**Fig 6:**
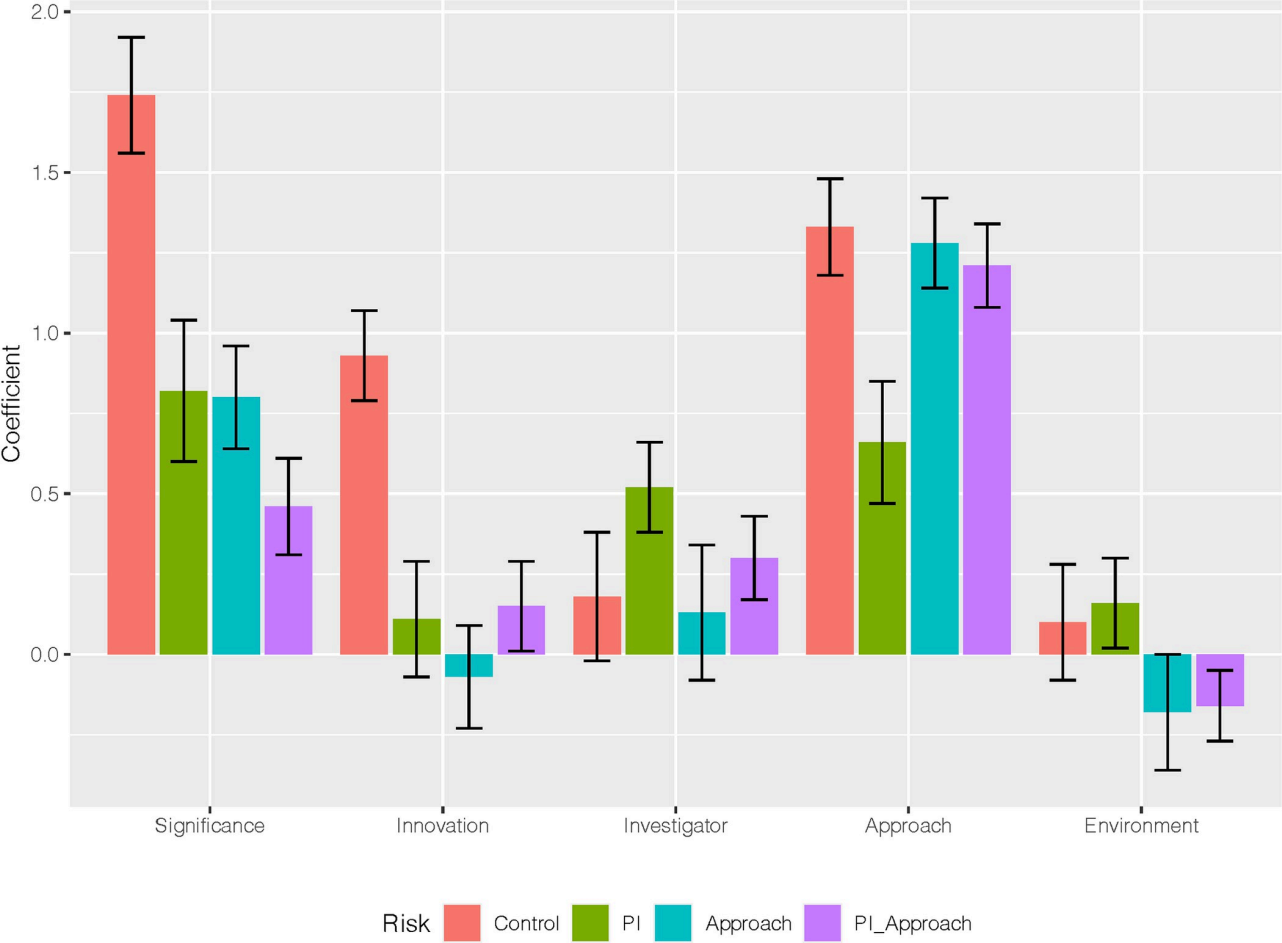
Contribution of criteria scores to overall scoring. Comparison of fit coefficients from four ordinal regression models of overall versus criteria scores. Four regression models (one for each OIS) were compared for relative importance of the 5 criteria scores in determining the overall score.

LOESS linear fits were used to visualize the relationships between overall and criteria scores for the different risk OISs in a series of jittered scatterplots (**Fig 5**). Overall scores for the risky OISs were less sensitive to criteria scores than was the control OIS, as is evidenced by the steeper slopes for the control OIS. The plots of overall score with approach are exceptions, where the slopes for all OIS risks are similar. The strength of these dependencies can be visualized via the coefficients of criteria predictors to overall score in simple ordinal regressions by OIS risk; **Fig 6** shows that significance and innovation criteria scores are stronger predictors of overall scores in the control OIS than in the risky OISs, while approach is a strong predictor of overall score for all of the OIS conditions.

### Control versus manipulated OIS scores

**Fig 7:**
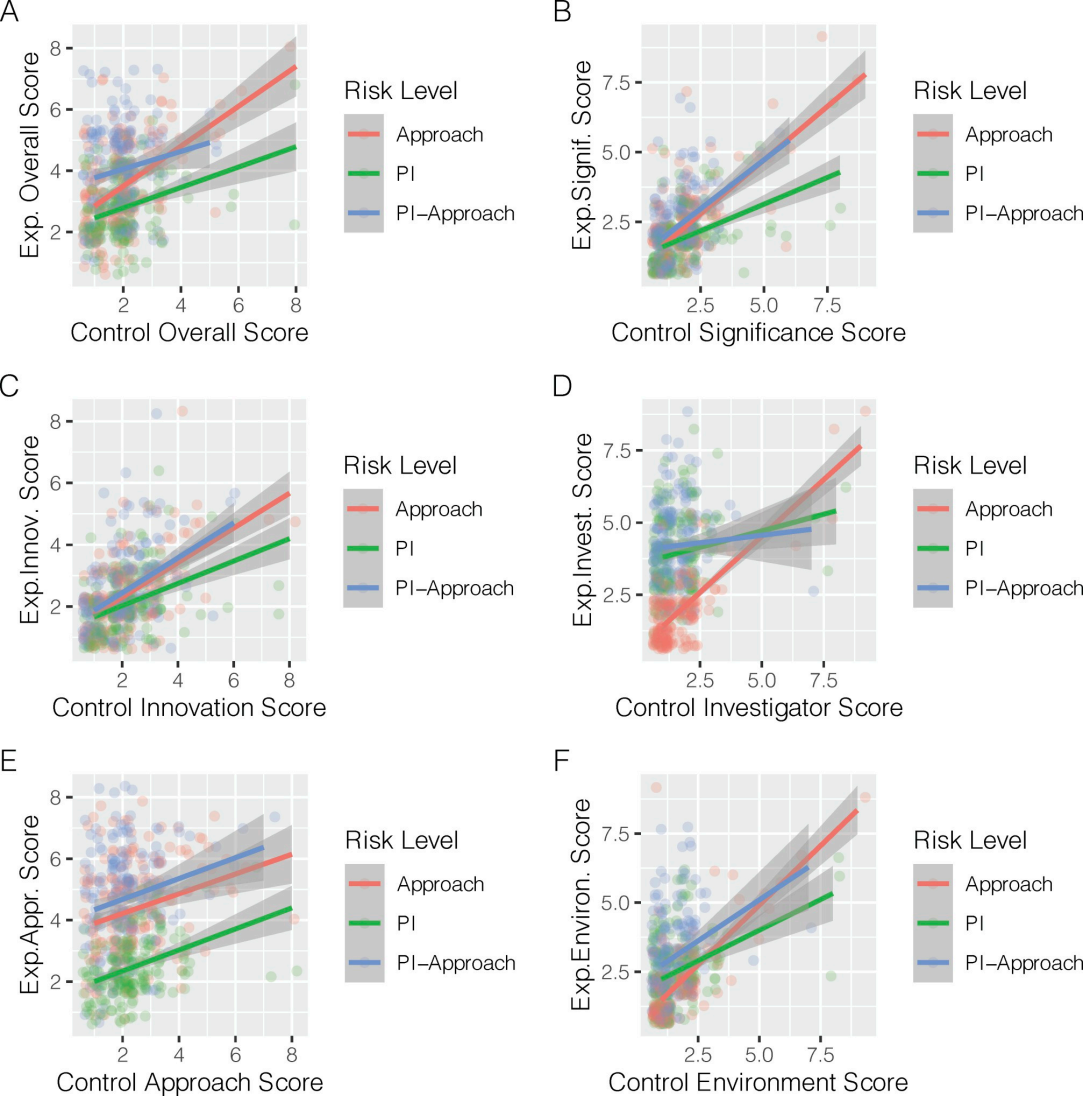
Control versus manipulated scores. Scatterplots and LOESS linear fits of scores from three manipulated OISs with one or more sources of risk compared with the control score for both the overall and all criteria scores. A. Overall; B. Significance; C. Innovation; D. Investigator; E. Approach; F. Environment.

Participant random effects are significant predictors of overall scores: one contributing factor may be the contrast of the manipulated and control OISs within participants; an examination of this relationship for different risk OISs is shown in **Fig 7**. These graphs indicate that manipulated and control scores are associated for overall and criteria scores, and suggest that, although these relationships change between risk scenarios, there is significant intra-rater consistency. However, across all the data, our measurements of Cronbach alpha of within-rater consistency between control and manipulated scores indicated the ratings of the manipulated components (the PI and approach) and the overall score are less consistent within raters than are the unmanipulated components (significance, innovation, environment; **S13 Table**).

## Discussion

### Risks in proposal evaluation

Risk was observed to be a strong predictor of overall and criteria scores, with which it interacted significantly. Risk impacted the scores for criteria that were not manipulated and were written as proposal strengths and assets (e.g., significance and innovation). As risk increased, the predictive power of these proposal assets towards the overall score diminished. In contrast, the approach criteria score was observed to strongly affect participants’ overall evaluations. These results suggest that reviewer evaluation of proposals’ risks dominates over that of proposal assets in OISs with proposal risk.

These results have broad implications for grant review and are aligned with previous studies that proposal scores are associated with the number of weaknesses in reviewers’ critiques but not with strengths [25]. Our findings suggest that evaluative rewards from innovative and potentially significant ideas are drowned out by evaluative penalties from risky aspects of proposals. These differences between rewards and risks may be partly due to the level of information inherent in these aspects of a proposal. For example, the evaluation of the longer-term benefits of research can be difficult. The OISs stated, “this work has high potential for translational impact,” but the specific impact was not described. More specific information was provided about risk and its impact on project success: “if the investigators find substantial toxicity or side effects in Aim 1, the other Aims can no longer be completed.” Information availability shapes decision making. The theory of bounded rationality, that people make rational decisions that are bounded by their expertise or knowledge [26,27], is relevant for peer review decisions [10,11]. Low myopic problem representation biases, where differences in information levels can cause an overweighting in comparative judgements, may also be pertinent [28–30]. More research is needed to apply these existing theories to peer review and explore how the clarity of translational impact may influence scoring for risky proposals.

### Risk impact

The source of risk (PI and/or approach) was an important factor in overall scores, with participants penalizing approach risks more than PI risks. The approach risk OIS described the impact of the risk to the experiments (i.e., dependency of Aims may prevent completion of the project). However, in the PI risk OIS, the inexperience of the PI was described as possibly affecting project completion, but how this risk would otherwise impact the study was not articulated. Thus, the information about the likelihood and potential impact of PI risk was less than approach risk, and was likely evaluated as relatively minor compared to the specific approach risk information. When both manipulations (PI and approach risk) are present in the same OIS, there may be a perceived increase in information, leading to the perception that these combined risks would impair project success.

### Risk intensity and scoring dispersion

As risk increased, the accurate detection of its presence by participants also increased, but so did the dispersion of participants’ scores, suggesting that proposal risk is an important source of inter-reviewer variability for projects of moderate-to-high quality. This relationship between risk and score distributions was found for overall scores and criteria scores. Pre-review orientations typically do not include guidance regarding how to translate risks into final scores. Reviewers may need training regarding risk acceptability and tolerance, and specific risk scenarios that could be evaluated positively, especially in distinguishing good from great proposals.

### Risk tolerance

The self-report questionnaire measure of risk tolerance, the NEO openness to experience scale [21], did not predict overall or most criteria scores, the exception being a very weak but significant predictor of the approach criteria score and its significant interaction with OIS risk. The low level of association between risk tolerance and OIS risk was surprising given the centrality of risk in our OIS manipulations. In the control OIS, risk tolerance was associated with more favorable approach scores, but this relationship largely disappeared in the manipulated OISs. Reviewer evaluation of approach in an outstanding proposal–as with our control OIS–may represent reviewer predisposition to risk. The literature suggests risk tolerance can be self-reported or revealed experimentally, and that the two types of measures can lead to different conclusions [31]. The approach score of an outstanding proposal could also simply reflect the level of leniency or stringency of participants as evaluators, despite our self-reported measure of leniency (see Methods) not being correlated with scores. Bias related to reviewers’ stringency has been reported for large studies of NIH review data [32]. In our study, this leniency effect depends on the riskiness of the OIS, as well as whether the component being scored was a manipulated component in the OIS. The least consistency between control and manipulated OIS scoring was in the approach score, despite the fact that the approach criteria was a key overall score-driving criteria in our and previous studies [20,19,33]. Understanding the interaction between evaluative stringency, sources of risk, and review criteria is an area for future investigation.

### Potential limitations

A limitation of this study is that evaluating proposals based on reading OISs is not representative of actual grant review, where scientists have access to the whole proposal, hear summaries from multiple assigned reviewers, and discuss the proposal before scoring. However, previous studies have shown that most unassigned reviewers’ scores are highly correlated with the assigned reviewers’ scores, which change minimally after discussion [34]. Another limitation is the low inter-rater agreement among participants, consistent with previous reports of grant review [7]; however, for all risk scenarios, the majority of reviewers in our study agree that the manipulated risk scenario should receive a worse score than the control. More research should focus on how reviewers translate this penalty into a numeric score and on developing review procedures which reduce variability due to this translation. Also, our response rates for this experiment were low, which could affect the generalizability of this work; however, these response rates are on par with similar previous surveys on peer review [35–37] Given the demographic similarity of our sample to NIH reviewers [23] and our inclusion criteria of review experience, this sample is likely to be highly representative of grant reviewers at NIH. Further, participants who started but did not complete the study may be attributable to limited review experience based on the data provided. Finally, our risk tolerance measure was not predictive of scores, and better alternatives may need to be created specific to peer review.

### Conclusions

This study found proposal risks–more than proposal strengths—to be a score-driving factor in the peer review of moderate- to high quality proposals. These results are of concern insofar as evaluations of highly innovative but risky proposals may be attenuated. Inter-reviewer score variability may depend on the perceived likelihood and impact of risks on project success and may represent an area of needed intervention for peer reviewers. More conversations in the scientific community about values and priorities in peer review would be beneficial, e.g., of acceptable levels of risk and potential failure in grant proposals, and how to appropriately balance the assessment of risks with assets. Ultimately, expectations of peer reviewers should be tempered with research on peer review processes and limitations.

## Supporting information

S1 Fig NEO openness. Risk Tolerance (NEO Openness Score) vs Approach Scores (LOESS plots).
(PDF)

S1 Table VIF analysis. VIF analysis for mixed ordinal regression of overall scoring data.
(PDF)

S2 Table Kruskal-Wallis. Kruskal-Wallis rank tests examining differences in participant scoring over the four risk scenarios: the control and the 3 manipulated OISs.
(PDF)

S3 Table Significance score regression comparisons. Significance Score–Multi-level ordinal regression models made with the reduced data set for direct comparison (n = 559).
(PDF)

S4 Table Significance final model. Cumulative Link Mixed Model of Significance Score fitted with the Laplace approximation from the total data set (605 participants).
(PDF)

S5 Table Innovation score regression comparisons. Innovation Score–Multi-level Ordinal Regression models made with the reduced data set for direct comparison (n = 559).
(PDF)

S6 Table Innovation final model. Cumulative Link Mixed Model of Innovation Score fitted with the Laplace approximation from the total data set (605 participants).
(PDF)

S7 Table Investigator score regression comparisons. Investigator Score–Multi-level Ordinal Regression models made with the reduced data set for direct comparison (n = 559).
(PDF)

S8 Table Investigator final model. Cumulative Link Mixed Model of Investigator Score fitted with the Laplace approximation from the total data set (605 participants).
(PDF)

S9 Table Approach score regression comparisons. Approach Score–Multi-level Ordinal Regression models made with the reduced data set for direct comparison (n = 559).
(PDF)

S10 Table Approach final model. Cumulative Link Mixed Model of Approach Score fitted with the Laplace approximation from the total data set (605 participants).
(PDF)

S11 Table Environment score regression comparisons. Environment Score–Multi-level Ordinal Regression models made with the reduced data set for direct comparison (n = 559).
(PDF)

S12 Table Environment final model. Cumulative Link Mixed Model of Environment Score fitted with the Laplace approximation from the total data set (605 participants).
(PDF)

S13 Table Cronbach’s alpha. Cronbach’s alpha and correlation of within-rater consistency between the rating of control and risk scenarios.
(PDF)

S1 File OIS text. Text of the four OIS impact statements.
(PDF)
