# Supplementary figures and images for: Peer review: Risk and risk tolerance
Source: PLoS One. 2022 Aug 26;17(8):e0273813. doi: 10.1371/journal.pone.0273813 (PMC9417194; doi:10.1371/journal.pone.0273813)

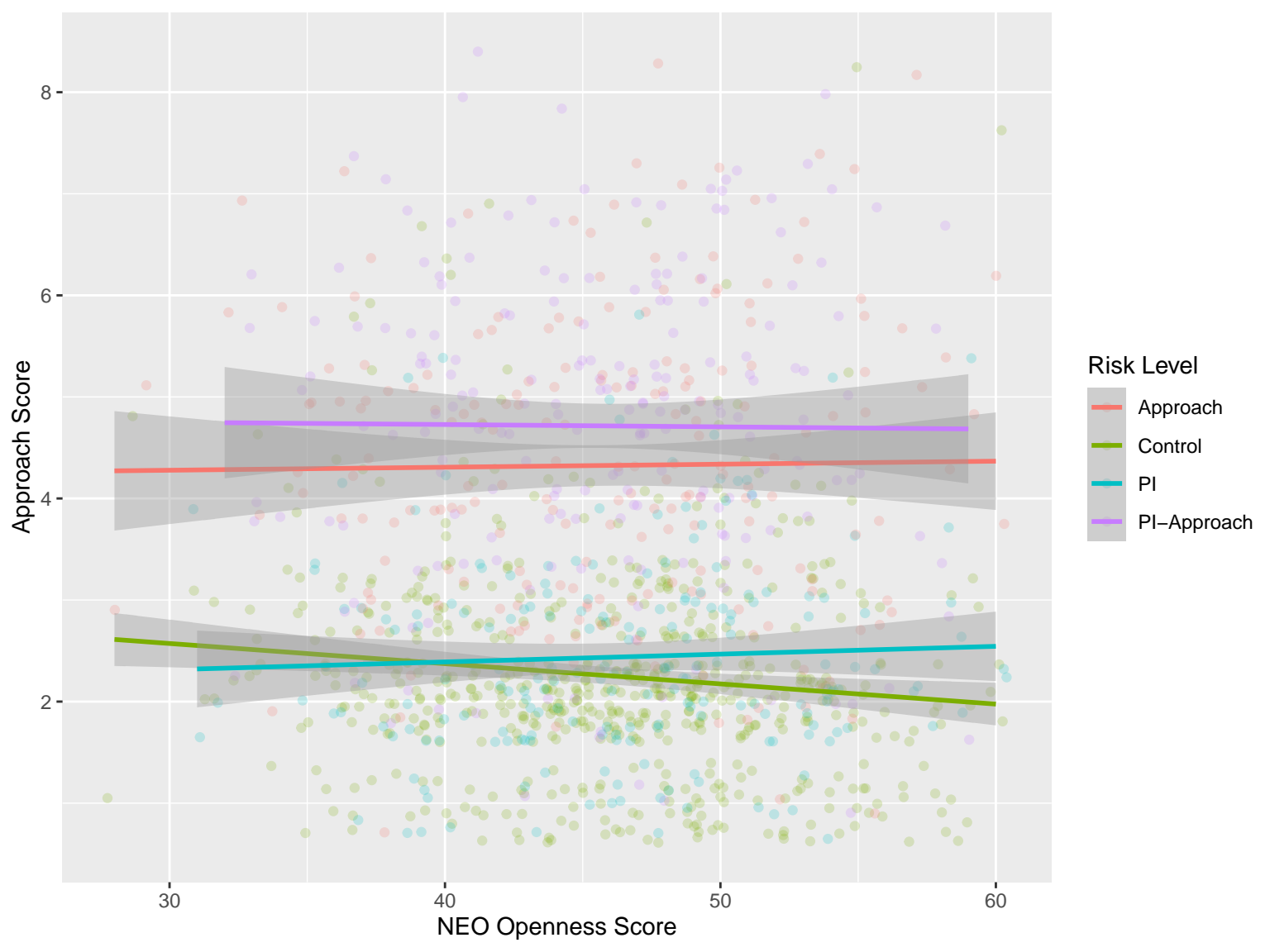

Supplement: S1 Fig — Risk Tolerance (NEO Openness Score) vs Approach Scores (LOESS plots). (PDF) [file pone.0273813.s001.pdf]
