# Supplementary material for: Peer review: Risk and risk tolerance
Source: PLoS One. 2022 Aug 26;17(8):e0273813. doi: 10.1371/journal.pone.0273813 (PMC9417194; doi:10.1371/journal.pone.0273813)
Supplement: S1 Table — VIF analysis for mixed ordinal regression of overall scoring data. (PDF) [file pone.0273813.s002.pdf]

S1 Table – **VIF Analysis.** VIF analysis for mixed ordinal regression of overall scoring data

Variable	GVIF	Df	$GVIF^{(1/(2*Df))}$
Risk	4.4	3	1.3
Gender	1.1	2	1.0
Race Ethnicity	1.4	1	1.2
English as a First Language	1.3	1	1.2
PhD	2.0	1	1.4
MD	2.0	1	1.4
Year Since Last Degree	1.1	1	1.1
Total Review Panels in the last 3 years	1.1	1	1.0
Research Similarity	1.1	1	1.0
Evaluative Predisposition	1.1	1	1.0
Risk Tolerance	1.1	1	1.1
Significance Score	2.0	1	1.4
Innovation Score	1.8	1	1.3
Investigator Score	4.0	1	2.0
Approach Score	2.6	1	1.6
Environment Score	2.4	1	1.5
