# Supplementary material for: Peer review: Risk and risk tolerance
Source: PLoS One. 2022 Aug 26;17(8):e0273813. doi: 10.1371/journal.pone.0273813 (PMC9417194; doi:10.1371/journal.pone.0273813)
Supplement: S2 Table — Kruskal-Wallis rank tests examining differences in participant scoring over the four risk scenarios: the control and the 3 manipulated OISs. (PDF) [file pone.0273813.s003.pdf]

S2 Table – **Kruskal-Wallis**. Kruskal-Wallis rank tests examining differences in participant scoring over the four risk scenarios: the control and the 3 manipulated OISs.

Score	Kruskal-Wallis Chi Square	p-value	Effect Size (eta^2)
Overall	437	<0.001	0.36
Significance	497	<0.001	0.06
Innovation	222	<0.001	0.02
Investigator	803	<0.001	0.52
Approach	609	<0.001	0.39
Environment	548	<0.001	0.21
