# Supplementary material for: Peer review: Risk and risk tolerance
Source: PLoS One. 2022 Aug 26;17(8):e0273813. doi: 10.1371/journal.pone.0273813 (PMC9417194; doi:10.1371/journal.pone.0273813)
Supplement: S3 Table — Significance Score–Multi-level ordinal regression models made with the reduced data set for direct comparison (n = 559). (PDF) [file pone.0273813.s004.pdf]

S3 Table – **Significance score regression comparisons.** Significance Score – Multi-level ordinal regression models made with the reduced data set for direct comparison (n=559).

Model	Variance Across Participants	Changes in 2LL (Previous Model)	Nagelkerke R^2
Baseline Across Participants	2.843	93.7**	---
Risk (R)	4.845	139.5**	0.13**
R + Demographic Variable Block (DV)	4.674	12.3	0.14**
R + DV + Research Similarity (RS)	4.577	3.9*	0.14**
R + DV + RS + Pre-disposition (PD)	4.585	2	0.14**
R + DV + RS + PD + Risk Preference (NEO)	4.548 (3.171, 6.173)	0.1	0.14**

* $p< 0.05$; ** $p<0.01$; 95% CI in parentheses; each successive model is compared to previous via -2LL (a fixed intercept model was used as baseline); Nagelkerke R^2 was calculated comparing to baseline model
