# Supplementary material for: Peer review: Risk and risk tolerance
Source: PLoS One. 2022 Aug 26;17(8):e0273813. doi: 10.1371/journal.pone.0273813 (PMC9417194; doi:10.1371/journal.pone.0273813)
Supplement: S4 Table — Cumulative Link Mixed Model of Significance Score fitted with the Laplace approximation from the total data set (605 participants). (PDF) [file pone.0273813.s005.pdf]

**S4 Table – Significance final model.** Cumulative Link Mixed Model of Significance Score fitted with the Laplace approximation from the total data set (605 participants).

| Term | Odds Ratio | 95% CI | p-value |
|---|---|---|---|
| Risk | | | |
| PI Risk | 1.80 | 1.19, 2.71 | 0.0052** |
| Approach Risk | 4.82 | 3.17, 7.33 | <0.0001*** |
| PI-Approach Risk | 8.31 | 5.36, 12.9 | <0.0001*** |
| Demographic Block | | | |
| Gender (Male) | 1.70 | 1.04, 2.77 | 0.0339* |
| Gender (Non-Binary) | 1.81 | 0.01, 334.20 | 0.8232 |
| Race Ethnicity (Non-White) | 1.59 | 0.86, 2.95 | 0.1396 |
| English as a First Language (Yes) | 1.17 | 0.67, 2.04 | 0.5871 |
| PhD (Yes) | 0.79 | 0.34, 1.83 | 0.5834 |
| MD (Yes) | 0.62 | 0.31, 1.25 | 0.1818 |
| Year Since Last Degree | 1.01 | 0.99, 1.04 | 0.3478 |
| Total Review Panels in the last 3 years | 0.99 | 0.97, 1.01 | 0.4121 |
| Research Similarity | 1.13 | 0.99, 1.29 | 0.0669 |
| Evaluative Predisposition | 1.14 | 0.95, 1.37 | 0.1545 |
| NEO Openness Scale | 0.97 | 0.77, 1.22 | 0.7908 |
| Threshold Coefficients | | | |
| 1\|2 | 0.89 | -0.68, 2.46 | 0.2648 |
| 2\|3 | 4.76 | 3.14, 6.39 | 0.0001*** |
| 3\|4 | 6.57 | 4.89, 8.26 | <0.0001*** |
| 4\|5 | 7.46 | 5.74, 9.18 | <0.0001*** |
| 5\|6 | 8.88 | 7.06, 10.71 | <0.0001*** |
| 6\|7 | 9.38 | 7.49, 11.27 | <0.0001*** |
| 7\|8 | 10.15 | 8.14, 12.17 | <0.0001*** |
| 8\|9 | 11.46 | 9.05, 13.87 | <0.0001*** |

* p< 0.05; ** p<0.01; *** p<0.001
