# Supplementary material for: Peer review: Risk and risk tolerance
Source: PLoS One. 2022 Aug 26;17(8):e0273813. doi: 10.1371/journal.pone.0273813 (PMC9417194; doi:10.1371/journal.pone.0273813)
Supplement: S5 Table — Innovation Score–Multi-level Ordinal Regression models made with the reduced data set for direct comparison (n = 559). (PDF) [file pone.0273813.s006.pdf]

S5 Table - Innovation score regression comparisons. Innovation Score – Multi-level Ordinal Regression models made with the reduced data set for direct comparison (n=559).

Model	Variance Across Participants	Changes in 2LL (Previous Model)	Nagelkerke R^2
Baseline Across Participants	3.061	113.6**	---
Risk (R)	3.505	44.4**	0.04**
R + Demographic Variable Block (DV)	3.240	30.4**	0.07**
R + DV + Research Similarity (RS)	3.223	1.4	0.07**
R + DV + RS + Pre-disposition (PD)	3.216	0.6	0.07**
R + DV + RS + PD + Risk Preference (NEO)	3.205 (2.251, 4.327)	0.9	0.07**

* p< 0.05; ** p<0.01; 95% CI in parentheses; each successive model is compared to previous via -2LL (a fixed intercept model was used as baseline); Nagelkerke R^2 was calculated comparing to baseline model
