# Supplementary material for: Peer review: Risk and risk tolerance
Source: PLoS One. 2022 Aug 26;17(8):e0273813. doi: 10.1371/journal.pone.0273813 (PMC9417194; doi:10.1371/journal.pone.0273813)
Supplement: S6 Table — Cumulative Link Mixed Model of Innovation Score fitted with the Laplace approximation from the total data set (605 participants). (PDF) [file pone.0273813.s007.pdf]

S6 Table - Innovation final model. Cumulative Link Mixed Model of Innovation Score fitted with the Laplace approximation from the total data set (605 participants).

Term	Odds Ratio	95% CI	p-value
Risk			
PI Risk	1.07	0.74, 1.57	0.7177
Approach Risk	2.35	1.61, 3.41	<0.0001***
PI-Approach Risk	2.72	1.87, 3.96	<0.0001***
Demographic Block			
Gender (Male)	1.80	1.18, 2.75	0.0067**
Gender (Non-Binary)	0.19	0.00, 18.91	0.4756
Race Ethnicity (Non-White)	1.51	0.89, 2.58	0.1283
English as a First Language (Yes)	1.78	1.09, 2.89	0.0201*
PhD (Yes)	1.80	0.87, 3.71	0.1140
MD (Yes)	0.92	0.50, 1.66	0.7699
Year Since Last Degree	1.02	1.00, 1.04	0.0612
Total Review Panels in the last 3 years	0.98	0.96, 1.00	0.0405*
Research Similarity	1.06	0.95, 1.19	0.2851
Evaluative Predisposition	1.07	0.91, 1.25	0.4232
NEO Openness Scale	0.91	0.74, 1.11	0.3462
Threshold Coefficients			
1|2	0.78	-0.57, 2.13	0.2559
2|3	3.87	2.48, 5.26	<0.0001***
3|4	5.94	4.50, 7.38	<0.0001***
4|5	7.19	5.69, 8.68	<0.0001***
5|6	8.72	7.11, 10.3	<0.0001***
6|7	9.57	7.84, 11.3	<0.0001***
7|8	9.76	7.99, 11.5	<0.0001***

* p< 0.05; ** p<0.01; *** p<0.001
