# Supplementary material for: Peer review: Risk and risk tolerance
Source: PLoS One. 2022 Aug 26;17(8):e0273813. doi: 10.1371/journal.pone.0273813 (PMC9417194; doi:10.1371/journal.pone.0273813)
Supplement: S7 Table — Investigator Score–Multi-level Ordinal Regression models made with the reduced data set for direct comparison (n = 559). (PDF) [file pone.0273813.s008.pdf]

S7 Table - Investigator score regression comparisons. Investigator Score – Multi-level Ordinal Regression models made with the reduced data set for direct comparison (n=559).

Model	Variance Across Participants	Changes in 2LL (Previous Model)	Nagelkerke R^2
Baseline Across Participants	1E-09	0	---
Risk (R)	1.192	856.5**	0.56**
R + Demographic Variable Block (DV)	1.145	10.2	0.56**
R + DV + Research Similarity (RS)	1.136	7.4	0.56**
R + DV + RS + Pre-disposition (PD)	1.134	0.5	0.57**
R + DV + RS + PD + Risk Preference (NEO)	1.136<br>(0.578, 1.880)	0.1	0.57**

* $p < 0.05$; ** $p < 0.01$; 95% CI in parentheses; each successive model is compared to previous via -2LL (a fixed intercept model was used as baseline); Nagelkerke R^2 was calculated comparing to baseline model
