# Supplementary material for: Peer review: Risk and risk tolerance
Source: PLoS One. 2022 Aug 26;17(8):e0273813. doi: 10.1371/journal.pone.0273813 (PMC9417194; doi:10.1371/journal.pone.0273813)
Supplement: S8 Table — Cumulative Link Mixed Model of Investigator Score fitted with the Laplace approximation from the total data set (605 participants). (PDF) [file pone.0273813.s009.pdf]

S8 Table - Investigator final model. Cumulative Link Mixed Model of Investigator Score fitted with the Laplace approximation from the total data set (605 participants).

Term	Odds Ratio	95% CI	p-value
Risk			
PI Risk	175.23	98.00, 313.34	<0.0001***
Approach Risk	2.69	1.86, 3.90	<0.0001***
PI-Approach Risk	234.53	128.60, 427.80	<0.0001***
Demographic Block			
Gender (Male)	1.42	1.02, 1.97	0.0352*
Gender (Non-Binary)	6.91	0.31, 156.49	0.2247
Race Ethnicity (Non-White)	0.97	0.64, 1.45	0.8657
English as a First Language (Yes)	0.92	0.64, 1.33	0.6538
PhD (Yes)	0.97	0.56, 1.70	0.9271
MD (Yes)	1.17	0.74, 1.85	0.4903
Year Since Last Degree	1.01	1.00, 1.03	0.1559
Total Review Panels in the last 3 years	1.00	0.98, 1.01	0.7835
Research Similarity	1.13	1.03, 1.23	0.0084**
Evaluative Predisposition	1.04	0.92, 1.18	0.4857
NEO Openness Scale	1.02	0.87, 1.19	0.8077
Threshold Coefficients			
1|2	1.53	0.47, 2.59	0.0047**
2|3	4.09	2.96, 5.22	<0.0001***
3|4	5.81	4.62, 7.01	<0.0001***
4|5	7.14	5.90, 8.39	<0.0001***
5|6	8.60	7.28, 9.91	<0.0001***
6|7	9.75	8.37, 11.1	<0.0001***
7|8	10.50	9.04, 11.9	<0.0001***
8|9	11.80	10.10, 13.60	<0.0001***

* p< 0.05; ** p<0.01; *** p<0.001
