# Supplementary material for: Peer review: Risk and risk tolerance
Source: PLoS One. 2022 Aug 26;17(8):e0273813. doi: 10.1371/journal.pone.0273813 (PMC9417194; doi:10.1371/journal.pone.0273813)
Supplement: S9 Table — Approach Score–Multi-level Ordinal Regression models made with the reduced data set for direct comparison (n = 559). (PDF) [file pone.0273813.s010.pdf]

S9 Table - Approach score regression comparisons. Approach Score – Multi-level Ordinal Regression models made with the reduced data set for direct comparison (n=559).

Model	Variance Across Participants	Changes in 2LL (Previous Model)	Nagelkerke R^2
Baseline Across Participants	3E-09	0	---
Risk (R)	1.083	605.6**	0.43**
R + Demographic Variable Block (DV)	1.037	6.7	0.44**
R + DV + Research Similarity (RS)	1.037	0	0.44**
R + DV + RS + Pre-disposition (PD)	1.036	0.1	0.44**
R + DV + RS + PD + Risk Preference (NEO)	1.028	2.5	0.44**
R + DV + RS + PD + NEO + Risk:NEO Interactions	1.085 (0.624, 1.673)	9.8*	0.44**

* $p < 0.05$; ** $p < 0.01$; 95% CI in parentheses; each successive model is compared to previous via -2LL (a fixed intercept model was used as baseline); Nagelkerke R^2 was calculated comparing to baseline model
