# Supplementary material for: Peer review: Risk and risk tolerance
Source: PLoS One. 2022 Aug 26;17(8):e0273813. doi: 10.1371/journal.pone.0273813 (PMC9417194; doi:10.1371/journal.pone.0273813)
Supplement: S10 Table — Cumulative Link Mixed Model of Approach Score fitted with the Laplace approximation from the total data set (605 participants). (PDF) [file pone.0273813.s011.pdf]

S10 Table - Approach final model. Cumulative Link Mixed Model of Approach Score fitted with the Laplace approximation from the total data set (605 participants).

Term	Odds Ratio	95% CI	p-value
Risk			
PI Risk	1.64	1.17, 2.29	0.0039**
Approach Risk	35.70	23.40, 54.50	<0.0001***
PI-Approach Risk	66.80	41.70, 107.00	<0.0001***
Demographic Block			
Gender (Male)	1.23	0.90, 1.66	0.1945
Gender (Non-Binary)	3.48	0.16, 75.60	0.4266
Race Ethnicity (Non-White)	1.22	0.83, 1.81	0.3100
English as a First Language (Yes)	1.18	0.83, 1.68	0.3474
PhD (Yes)	1.25	0.73, 2.14	0.4075
MD (Yes)	1.06	0.68, 1.65	0.7870
Year Since Last Degree	1.00	0.98, 1.01	0.7661
Total Review Panels in the last 3 years	1.00	0.99, 1.01	0.8450
Research Similarity	0.99	0.91, 1.08	0.8671
Evaluative Predisposition	1.03	0.92, 1.16	0.6018
NEO Openness Scale	0.73	0.60, 0.89	0.0016**
Risk (PI Only): NEO	1.47	1.04, 2.08	0.0287*
Risk (Approach Only): NEO	1.41	1.03, 1.93	0.0316*
Risk (PI-Approach): NEO	1.42	1.00, 2.02	0.0491*
Threshold Coefficients			
1|2	-1.11	-2.11, -0.11	0.0302*
2|3	1.62	0.62, 2.63	0.0015**
3|4	3.26	2.22, 4.29	<0.0001***
4|5	4.42	3.35, 5.48	<0.0001***
5|6	5.74	4.63, 6.85	<0.0001***
6|7	6.94	5.78, 8.09	<0.0001***
7|8	8.85	7.52, 10.2	<0.0001***

* p< 0.05; ** p<0.01; *** p<0.001
