# Supplementary material for: Peer review: Risk and risk tolerance
Source: PLoS One. 2022 Aug 26;17(8):e0273813. doi: 10.1371/journal.pone.0273813 (PMC9417194; doi:10.1371/journal.pone.0273813)
Supplement: S11 Table — Environment Score–Multi-level Ordinal Regression models made with the reduced data set for direct comparison (n = 559). (PDF) [file pone.0273813.s012.pdf]

S11Table - Environment score regression comparisons. Environment Score – Multi-level Ordinal Regression models made with the reduced data set for direct comparison (n=559).

Model	Variance Across Participants	Changes in 2LL (Previous Model)	Nagelkerke R^2
Baseline Across Participants	0.5367	11.2**	---
Risk (R)	3.744	389.7**	0.32**
R + Demographic Variable Block (DV)	3.55	18.2*	0.33**
R + DV + Research Similarity (RS)	3.433	11.3**	0.33**
R + DV + RS + Pre-disposition (PD)	3.427	1.3	0.34 **
R + DV + RS + PD + Risk Preference (NEO)	3.41 (2.212, 4.867)	0.8	0.34**

* $p< 0.05$; ** $p<0.01$; 95% CI in parentheses; each successive model is compared to previous via -2LL (a fixed intercept model was used as baseline); Nagelkerke R^2 was calculated comparing to baseline model
