# Supplementary material for: Peer review: Risk and risk tolerance
Source: PLoS One. 2022 Aug 26;17(8):e0273813. doi: 10.1371/journal.pone.0273813 (PMC9417194; doi:10.1371/journal.pone.0273813)
Supplement: S12 Table — Cumulative Link Mixed Model of Environment Score fitted with the Laplace approximation from the total data set (605 participants). (PDF) [file pone.0273813.s013.pdf]

S12 Table - Environment final model. Cumulative Link Mixed Model of Environment Score fitted with the Laplace approximation from the total data set (605 participants).

Term	Odds Ratio	95% CI	p-value
Risk			
PI Risk	18.27	11.50, 29.01	<0.0001***
Approach Risk	4.11	2.72, 6.22	<0.0001***
PI-Approach Risk	47.77	28.21, 80.87	<0.0001***
Demographic Block			
Gender (Male)	0.96	0.62, 1.50	0.8630
Gender (Non-Binary)	5.19	0.05, 557	0.4898
Race Ethnicity (Non-White)	1.36	0.77, 2.39	0.2915
English as a First Language (Yes)	1.78	1.06, 2.99	0.0281*
PhD (Yes)	0.98	0.46, 2.10	0.9661
MD (Yes)	1.22	0.65, 2.29	0.5316
Year Since Last Degree	1.03	1.01, 1.05	0.0097**
Total Review Panels in the last 3 years	0.99	0.97, 1.00	0.1316
Research Similarity	1.22	1.08, 1.38	0.0015**
Evaluative Predisposition	1.11	0.94, 1.31	0.2262
NEO Openness Scale	0.91	0.74, 1.13	0.3976
Threshold Coefficients			
1|2	3.07	1.62, 4.53	<0.0001***
2|3	5.93	4.39, 7.47	<0.0001***
3|4	7.64	6.03, 9.25	<0.0001***
4|5	8.79	7.13, 10.45	<0.0001***
5|6	9.73	8.02, 11.44	<0.0001***
6|7	10.65	8.87, 12.43	<0.0001***
7|8	11.37	9.51, 13.24	<0.0001***
8|9	12.45	10.34, 14.56	<0.0001***

* p< 0.05; ** p<0.01; *** p<0.001
