# Supplementary material for: Peer review: Risk and risk tolerance
Source: PLoS One. 2022 Aug 26;17(8):e0273813. doi: 10.1371/journal.pone.0273813 (PMC9417194; doi:10.1371/journal.pone.0273813)
Supplement: S13 Table — Cronbach’s alpha and correlation of within-rater consistency between the rating of control and risk scenarios. (PDF) [file pone.0273813.s014.pdf]

S13 Table – Cronbach's alpha. Cronbach's alpha and correlation of within-rater consistency between the rating of control and risk scenarios

Variable	Correlation	N	Cronbach's Alpha
Overall	0.297	603	0.435
Significance	0.517	601	0.679
Innovation	0.467	601	0.636
Investigator	0.247	601	0.356
Approach	0.193	601	0.298
Environment	0.429	601	0.569
