# Supplementary material for: Peer review: Risk and risk tolerance
Source: PLoS One. 2022 Aug 26;17(8):e0273813. doi: 10.1371/journal.pone.0273813 (PMC9417194; doi:10.1371/journal.pone.0273813)
Supplement: S1 File — Text of the four OIS impact statements. (PDF) [file pone.0273813.s015.pdf]

APPENDIX

Overall Impact Statements

Instructions: You are an unassigned reviewer who hears the following summary from an assigned reviewed of an NIH R01 application. In this scenario, you do not have the ability to reference the original proposal or discuss the application with the assigned or other reviewers. Please score the overall impact and also significance, innovation, investigator, approach and environment. You may consult the score guidance table below.

(***Note***: a participant will receive OIS#1 and one other OIS randomly selected from #2-#7. Each OIS will appear on a different page. The score guidance table below will appear on each page for reference.)

Overall Impact or Criterion Strength	Score	Descriptor
High	1	Exceptional
	2	Outstanding
	3	Excellent
Medium	4	Very Good
	5	Good
	6	Satisfactory
Low	7	Fair
	8	Marginal
	9	Poor

Overall Impact Statement #1 (CONTROL)

Treatment of disease J, a devastating illness that affects the lives of millions of people worldwide, is limited to minimizing symptoms, as no efficacious therapy has been discovered. The stated aims of this proposal are to test molecule (M), which has had recent success in inhibiting the effects of disease J in an in vitro model. Except for the PI's very promising pilot data, M is untested in vivo for potential therapeutic efficacy in an animal model of J. The proposed studies also examine if M in combination therapy with molecule K can result in reduced therapeutic dosages to minimize potential toxicities and increase the likelihood of their use in humans. The investigation of molecule K is exciting given its potential for cross-sensitive interactions with molecule M and even if no interactions are observed, will likely provide important information about the signaling pathways involved in the pathogenesis of J. The innovative animal model was developed and piloted for this proposal to represent the clinical manifestations of disease J in humans. This potentially synergistic treatment is highly promising clinically, and these well-constructed studies will provide crucial data for the translation of in vitro findings to novel therapeutic applications. The proposal reflected a realistic appraisal of the limitations of preclinical models, and described preparatory steps for a Phase I trial. The project fits well within the scope of the funding opportunity. An excellent literature review of relevant mechanistic studies, and strong preliminary studies in rats, suggest a high feasibility to this work. Additional strengths include a compelling rationale for the need of these studies, strong methodology presented in good detail, the inclusion of appropriate controls, an excellent data analysis section, and a robust discussion of pitfalls and alternate approaches. The scientific environment at Q University is outstanding. The PI is an experienced investigator (particularly in animal model studies) with an impressive publication and funding record in this area who is uniquely positioned to conduct this work. The team has the appropriate expertise and seems to be ideally suited to conduct this study. Overall, there are considerable strengths to this exciting application, and only negligible weaknesses. This important research is necessary to move this potential therapeutic forward, and if successful will be highly significant.

Overall Impact: (1-9 on each)
Significance:
Innovation:
Investigator:
Approach:
Environment:

Overall Impact Statement #2 (High Risk Approach, Low Risk Investigator- Male)

The purpose of this proposal is to target the X pathway, which is involved in the Y response, and may be associated with the development of Disease D. Disease D is associated with substantial morbidity among those affected and is increasing in prevalence. The investigators propose to create a novel mimetic inhibitor to the XY interaction, and then using an experimental rodent model of D, test whether there is a reduction in D-related outcomes. Potential therapeutics for D are almost non-existent and little is known of the underlying mechanisms. Thus, this work has high potential for translational impact for a suffering population with few clinical options. It is responsive to the mission of the research funder, is highly innovative, and the results could be very significant. The proposal is very well written and has a logical research plan. These studies, in theory, are highly feasible given the laboratory's experience, technical prowess, and the excellent scientific environment. The PI, James Smith, is a full professor at Q University; he has previously had federally-funded grants, and he is well published in the area. Strengths of this proposal also include the excellent team and infrastructure available for completing the project, and the tremendous translational potential of these studies. The primary weakness of this proposal is the potential risk, as pilot studies of XY suppression in the rodent model of D are lacking. For instance, the inhibition of XY may have adverse effects that would preclude the successful completion of these studies. The potential for adverse effects is concerning as the X pathway may have some indirect involvement with Z in the rat model, which may produce harmful effects if inhibited. The investigators do mention this possibility, although they do not provide preliminary data. If the project is funded they plan to conduct a dose-response experiment in Aim 1, looking for such effects. However, if the investigators find substantial toxicity or side effects in Aim 1, the other Aims can no longer be completed. Despite this weakness, the study design is otherwise very strong and the potential for impact is extremely high. Overall, this reviewer felt this is a high risk, potentially high reward proposal.

Overall Impact: (1-9 on each)
Significance:
Innovation:
Investigator:
Approach:
Environment:

Overall Impact Statement #3 (Low Risk Approach, High Risk Investigator- Male)

The purpose of this proposal is to target the X pathway, which is involved in the Y response and may be associated with the development of Disease (D). Disease D is associated with substantial morbidity among those affected and is increasing in prevalence. The investigators propose to create a novel mimetic inhibitor to the XY interaction, and then using an experimental rodent model of D, test whether there is a reduction in D-related outcomes. Potential therapeutics for D are almost non-existent and little is known of the underlying mechanisms. Thus, this work has a high potential for translational impact for a suffering population with few clinical options. It is responsive to the mission of the research funder, is highly innovative, and the results could be very significant. The proposal is very well written and has a logical research plan. These studies, in theory, are highly feasible given the excellent environment. The PI, James Smith, is a new investigator and has a relatively junior position at Q University. In addition, there were strong preliminary data, supporting the feasibility of the proposed work. An important strength of this proposal is the tremendous translational potential of these studies. The primary weakness of this proposal is the potential risk associated with the PI's limited experience in leading independent research. His affiliate position at Q University is a semi-independent one that allows him to apply to this funding opportunity. His publication record is somewhat limited, only having one publication in the field of D. However, he did include a letter of institutional commitment from the university, which provides support for his ability to complete these studies. Still, it is not completely clear the investigator has the experience necessary to complete the aims. Despite this weakness, the study design is very strong and the potential for impact is extremely high. Overall, this reviewer felt this is a high risk, potentially high reward proposal.

Overall Impact: (1-9 on each)
Significance:
Innovation:
Investigator:
Approach:
Environment:

Overall Impact Statement #4 (High Risk Approach, High Risk Investigator- Male)

The purpose of this proposal is to target the X pathway, which is involved in the Y response, and may be associated with the development of Disease D. Disease D is associated with substantial morbidity among those affected and is increasing in prevalence. The investigators propose to create a novel mimetic inhibitor to the XY interaction, and then using an experimental rodent model of D, to test whether there is a reduction in D-related outcomes. Potential therapeutics for D are almost non-existent and little is known of the underlying mechanisms. Thus, this work has high potential for translational impact for a suffering population with few clinical options. It is responsive to the mission of the research funder, is highly innovative, and the results could be very significant. The proposal is very well written and has a logical research plan. These studies, in theory, are highly feasible given the excellent environment. The PI, James Smith, is a new investigator and has a relatively junior position at Q University. An important weakness of this proposal is its potential risk, as pilot studies of XY suppression in the rodent model of D are lacking. For instance, the inhibition of XY may have adverse effects that would preclude the successful completion of these studies. The potential for adverse effects is concerning as the X pathway may have some indirect involvement with Z in the rat model, which may produce harmful effects if inhibited. The investigators do mention this possibility, although they do not provide preliminary data. If the project is funded they plan to conduct a dose-response experiment in Aim 1, looking for such effects. However, if the investigators find substantial toxicity or side effects in Aim 1, the other Aims can no longer be completed. An additional weakness of this proposal is the potential risk associated with the PI's limited experience in leading independent research. His affiliate position at Q University is a semi-independent one that allows him to apply to this funding opportunity. His publication record is somewhat limited, only having one publication in the field of D. However, he did include a letter of institutional commitment from the university, which provides support for his ability to complete these studies. Still, it is not completely clear the investigator has the experience necessary to complete the aims. Despite these weaknesses, the study design is otherwise very strong and the potential for impact is extremely high. Overall, this reviewer felt this is a high risk, potentially high reward proposal.

Overall Impact: (1-9 on each)
Significance:
Innovation:
Investigator:
Approach:
Environment:

Overall Impact Statement #5 (High Risk Approach, Low Risk Investigator- Female)

The purpose of this proposal is to target the X pathway, which is involved in the Y response, and may be associated with the development of Disease D. Disease D is associated with substantial morbidity among those affected and is increasing in prevalence. The investigators propose to create a novel mimetic inhibitor to the XY interaction, and then using an experimental rodent model of D, test whether there is a reduction in D-related outcomes. Potential therapeutics for D are almost non-existent and little is known of the underlying mechanisms. Thus, this work has high potential for translational impact for a suffering population with few clinical options. It is responsive to the mission of the research funder, is highly innovative, and the results could be very significant. The proposal is very well written and has a logical research plan. These studies, in theory, are highly feasible given the laboratory's experience, technical prowess, and the excellent scientific environment. The PI, Mary Smith, is a full professor at Q University; she has previously had federally-funded grants, and she is well published in the area. Strengths of this proposal also include the excellent team and infrastructure available for completing the project, and the tremendous translational potential of these studies. The primary weakness of this proposal is the potential risk, as pilot studies of XY suppression in the rodent model of D are lacking. For instance, the inhibition of XY may have adverse effects that would preclude the successful completion of these studies. The potential for adverse effects is concerning as the X pathway may have some indirect involvement with Z in the rat model, which may produce harmful effects if inhibited. The investigators do mention this possibility, although they do not provide preliminary data. If the project is funded they plan to conduct a dose-response experiment in Aim 1, looking for such effects. However, if the investigators find substantial toxicity or side effects in Aim 1, the other Aims can no longer be completed. Despite this weakness, the study design is otherwise very strong and the potential for impact is extremely high. Overall, this reviewer felt this is a high risk, potentially high reward proposal.

Overall Impact: (1-9 on each)
Significance:
Innovation:
Investigator:
Approach:
Environment:

Overall Impact Statement #6 (Low Risk Approach, High Risk Investigator- Female)

The purpose of this proposal is to target the X pathway, which is involved in the Y response and may be associated with the development of Disease (D). Disease D is associated with substantial morbidity among those affected and is increasing in prevalence. The investigators propose to create a novel mimetic inhibitor to the XY interaction, and then using an experimental rodent model of D, test whether there is a reduction in D-related outcomes. Potential therapeutics for D are almost non-existent and little is known of the underlying mechanisms. Thus, this work has a high potential for translational impact for a suffering population with few clinical options. It is responsive to the mission of the research funder, is highly innovative, and the results could be very significant. The proposal is very well written and has a logical research plan. These studies, in theory, are highly feasible given the excellent environment. The PI, Mary Smith, is a new investigator and has a relatively junior position at Q University. In addition, there were strong preliminary data, supporting the feasibility of the proposed work. An important strength of this proposal is the tremendous translational potential of these studies. The primary weakness of this proposal is the potential risk associated with the PI's limited experience in leading independent research. Her affiliate position at Q University is a semi-independent one that allows her to apply to this funding opportunity. Her publication record is somewhat limited, only having one publication in the field of D. However, she did include a letter of institutional commitment from the university, which provides support for her ability to complete these studies. Still, it is not completely clear the investigator has the experience necessary to complete the aims. Despite this weakness, the study design is very strong and the potential for impact is extremely high. Overall, this reviewer felt this is a high risk, potentially high reward proposal.

Overall Impact: (1-9 on each)
Significance:
Innovation:
Investigator:
Approach:
Environment:

Overall Impact Statement #7 (High Risk Approach, High Risk Investigator- Female)

The purpose of this proposal is to target the X pathway, which is involved in the Y response, and may be associated with the development of Disease D. Disease D is associated with substantial morbidity among those affected and is increasing in prevalence. The investigators propose to create a novel mimetic inhibitor to the XY interaction, and then using an experimental rodent model of D, to test whether there is a reduction in D-related outcomes. Potential therapeutics for D are almost non-existent and little is known of the underlying mechanisms. Thus, this work has high potential for translational impact for a suffering population with few clinical options. It is responsive to the mission of the research funder, is highly innovative, and the results could be very significant. The proposal is very well written and has a logical research plan. These studies, in theory, are highly feasible given the excellent environment. The PI, Mary Smith, is a new investigator and has a relatively junior position at Q University. An important weakness of this proposal is its potential risk, as pilot studies of XY suppression in the rodent model of D are lacking. For instance, the inhibition of XY may have adverse effects that would preclude the successful completion of these studies. The potential for adverse effects is concerning as the X pathway may have some indirect involvement with Z in the rat model, which may produce harmful effects if inhibited. The investigators do mention this possibility, although they do not provide preliminary data. If the project is funded they plan to conduct a dose-response experiment in Aim 1, looking for such effects. However, if the investigators find substantial toxicity or side effects in Aim 1, the other Aims can no longer be completed. An additional weakness of this proposal is the potential risk associated with the PI's limited experience in leading independent research. Her affiliate position at Q University is a semi-independent one that allows her to apply to this funding opportunity. Her publication record is somewhat limited, only having one publication in the field of D. However, she did include a letter of institutional commitment from the university, which provides support for her ability to complete these studies. Still, it is not completely clear the investigator has the experience necessary to complete the aims. Despite these weaknesses, the study design is otherwise very strong and the potential for impact is extremely high. Overall, this reviewer felt this is a high risk, potentially high reward proposal.

Overall Impact: (1-9 on each)
Significance:
Innovation:
Investigator:
Approach:
Environment:
